# NoxO1 promotes endosome formation and reduces intracellular vesicle processing

**DOI:** 10.1016/j.redox.2025.103973

**Published:** 2025-12-12

**Authors:** Maureen Hebchen, Falk Herwig, Tim Schader, Manuela Spaeth, Niklas Müller, Katrin Schröder

**Affiliations:** a Institute for Cardiovascular Physiology, Goethe University Frankfurt, Germany; b Dept of Physics & Astronomy, University of Victoria, Canada; c German Center of Cardiovascular Research (DZHK), Partner site RheinMain, Frankfurt, Germany

**Keywords:** NoxO1, Erbin, EGFR, TFEB, Lysosome biogenesis, Endosomal trafficking

## Abstract

NADPH oxidase organizer 1 (NoxO1) is known as a scaffold cytoplasmic subunit of the reactive oxygen species (ROS) forming Nox1 complex. We previously identified an interaction between NoxO1 and Erbin, a cytosolic scaffold protein that associates with Epidermal Growth Factor Receptor (EGFR), but its ROS-independent roles remain poorly understood.

Here, we demonstrate that NoxO1 overexpression remodels the endolysosomal system by expanding early endosomes and lysosomes. A calibrated six-compartment ordinary differential equation model of EGFR trafficking predicts a slowed down intracellular trafficking: NoxO1 overexpression increased internalization rates by 14 % while reducing degradative sorting by 48 %, lysosomal transfer by 24 %, and final degradation by 41 %. Using fluorescent cargo (EGF and BSA), we confirmed enhanced internalization and cargo accumulation in lysosomes, supporting the idea of prolonged lysosomal retention in NoxO1 overexpressing cells. Mechanistically, NoxO1 activated transcription factor EB (TFEB), the master regulator of lysosomal biogenesis, in an Erbin-dependent but ROS independent manner. Proximity ligation assays revealed spatial association of NoxO1, Erbin, EGFR, and TFEB, suggesting a multi-protein regulatory complex. Genetic ablation of Erbin abolished NoxO1-induced increases in early endosome (EEA1) and lysosome (LAMP1) markers, confirming Erbin's essential role.

In conclusion, via its interaction with Erbin NoxO1 promotes activation of TFEB, contributes to lysosome formation while delaying cargo degradation.

## Highlights

• NoxO1 increases early endosome and lysosome abundance in cultured cells.
• NoxO1 slows endosomal trafficking and increases lysosomal cargo retention.
• NoxO1 activates TFEB via Erbin, expanding the lysosomal/CLEAR program.
• Minimal contribution of NOX1-derived ROS under the tested conditions.
• A calibrated trafficking model explains the observed compartment shifts.

## Graphical abstract

**Figure:**
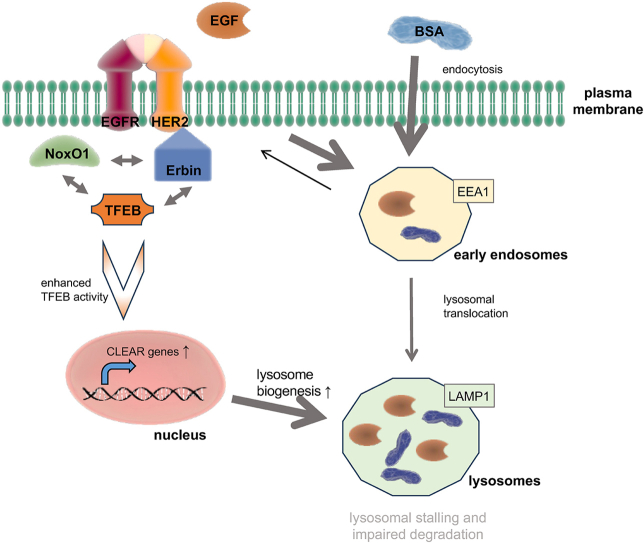

## 1 Introduction

NADPH oxidase organizer 1 (NoxO1) and Nox activator 1 (NoxA1) are known as cytosolic subunits of the Nox1 centered NADPH oxidase (Nox1) [1]. As part of this complex, NoxO1 activates the formation of reactive oxygen species (ROS). In many cells NoxO1 expression exceeds that of Nox1 and NoxA1, and its expression level determines the amount of ROS produced by the Nox1-centered NADPH oxidase [2]. NoxO1 contributes to formation of otoconia [3], and differentiation of gut enterocytes [4]. In the vascular system, a role of NoxO1 has been described for angiogenesis and atherosclerosis [5,6]. The role of NoxO1-mediated ROS formation in these processes, except for its role in angiogenesis, remains uncertain. NoxO1 localizes to cell membranes and contains several domains that facilitate protein-protein interactions, such as a proline-rich region (PRR) and two Src homology 3 (SH3) domains [7]. Recently, we identified Erbin as an interaction partner of NoxO1. Erbin interacts with the epithelial growth factor receptor (EGFR) and prevents its downstream signaling. Enhancing the association of Erbin and EGFR by overexpressing NoxO1 delays EGFR signaling, while knocking out NoxO1 increases it [8]. These findings suggest an ROS-independent aspect of NoxO1 function.

The EGFR belongs to the ErbB1-4 family of transmembrane tyrosine kinases, which play essential roles in cellular processes such as proliferation, survival, and migration. [9,10]. Binding of one of 14 ligands to a receptor family member, results in the formation of homodimers or heterodimers, followed by the autophosphorylation of the cytoplasmic kinase domains and subsequent internalization. Additionally, in the absence of EGF, EGFR is internalized and recycled in a steady state [11]. Both ligand-bound and unoccupied EGFR are internalized and delivered to early endosomes by a cell-specific endocytosis machinery [12,13]. In fact, endocytosis is a cell- and context-specific process. It is not only responsible for the internalization of growth factors and their receptors, but also for macromolecules such as low-density lipoprotein (LDL) and bovine serum albumin (BSA) [[14], [15], [16]]. The endosomal network has been modeled as either a stable pool of pre-formed organelles with a defined identity or a dynamic formation of evolving organelles [[17], [18], [19]]. Once found in early endosomes, the cargo is destined to recycling, degradation or transport to other organelles [20]. In this context, Ras-related proteins (Rab) serve as markers to identify endosomes and regulate maturation, cargo processing and endosomal signaling [21].

In this study, BSA and EGFR served as well-established model cargos to investigate the effect of NoxO1 on endosomal trafficking.

## 2 Results

### 2.1 NoxO1 formation of endosomal trafficking organelles

**Fig. 1:**
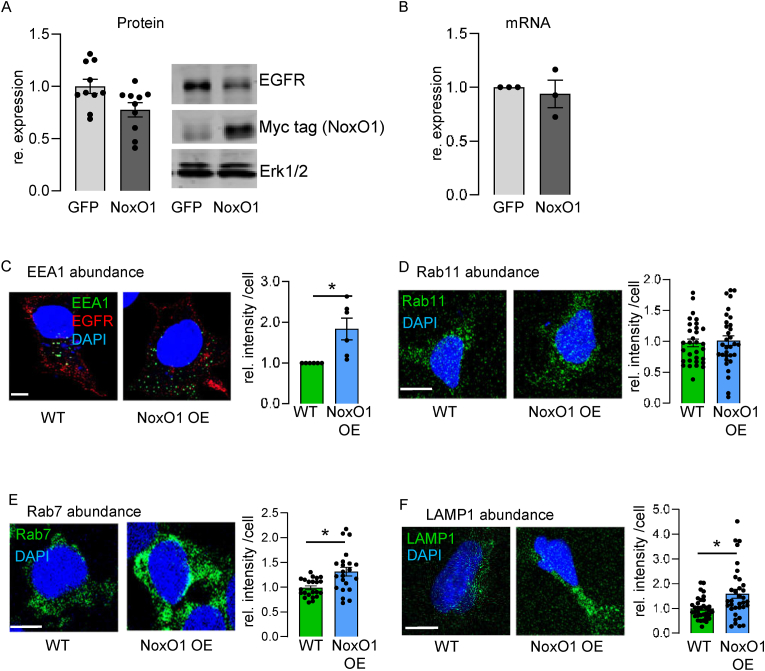
NoxO1 augments early endosome formation and degradative routes in Hek293. (A&B) Native Hek293 cells overexpressing GFP or NoxO1 (A: Western blot with statistics and B: qPCR), n = 10 and 3; ∗p < 0.05, *t*-test. Mean ± SEM; (C–F) Cells without (WT) and with constitutive overexpression of NoxO1 (NoxO1 OE); Representative images and statistics for (C) EEA1, (D) the recycling endosome marker Ras-related protein 11 (Rab11), (E) Ras-related protein 7 (Rab 7); (F) Lysosomal associated membrane protein 1 (LAMP1) as markers for late endosomes and lysosomes. Representative images, scale bar = 10 μm, Mean ± SEM, n = 3–4; ∗p < 0.05 in two-sided student's *t*-test for each condition. Mean ± SEM.

From earlier studies we know NoxO1 delays EGFR signaling [8]. Interestingly, overexpression of NoxO1 little but consistently reduces total epidermal growth factor receptor (EGFR) protein abundance (Fig. 1A) without affecting its mRNA expression (Fig. 1B). In order to make sure this is not an effect of overexpression of a random protein, we utilized GFP as a control. NoxO1 overexpression does not induce classical autophagy (Supplemental Fig. S1A) but still reduces the expression of a random protein such as a luciferase (Supplemental Fig. S1B), when compared to GFP overexpressing cells.

Internalized cargo must pass through other endosomal compartments on its way to the lysosome, beginning with the formation of early endosomes, the first sorting station for internalized cargo [21]. NoxO1 overexpression augmented Early Endosome Antigen 1 (EEA1) expression, which we interpreted as an elevation in early endosome formation (Fig. 1C). Once formed, early endosomes undergo sorting, which results in recycling or degradation of the cargo [22]. Rab11, a marker of recycling endosomes [22] remains unchanged (Fig. 1D). In contrast, NoxO1 overexpression enforced the expression of late endosomal and lysosomal markers, such as Rab7 (Fig. 1E) and Lysosomal-associated membrane protein 1 (LAMP1) (Fig. 1F), respectively. Inhibiting lysosomal degradation with chloroquine prevented the reduction of epidermal growth factor receptor (EGFR) in NoxO1 overexpressing cells (Supplemental Fig. S2).

### 2.2 Simulation of EGFR intracellular trafficking suggest reduced inter-organelle trafficking

**Fig. 2:**
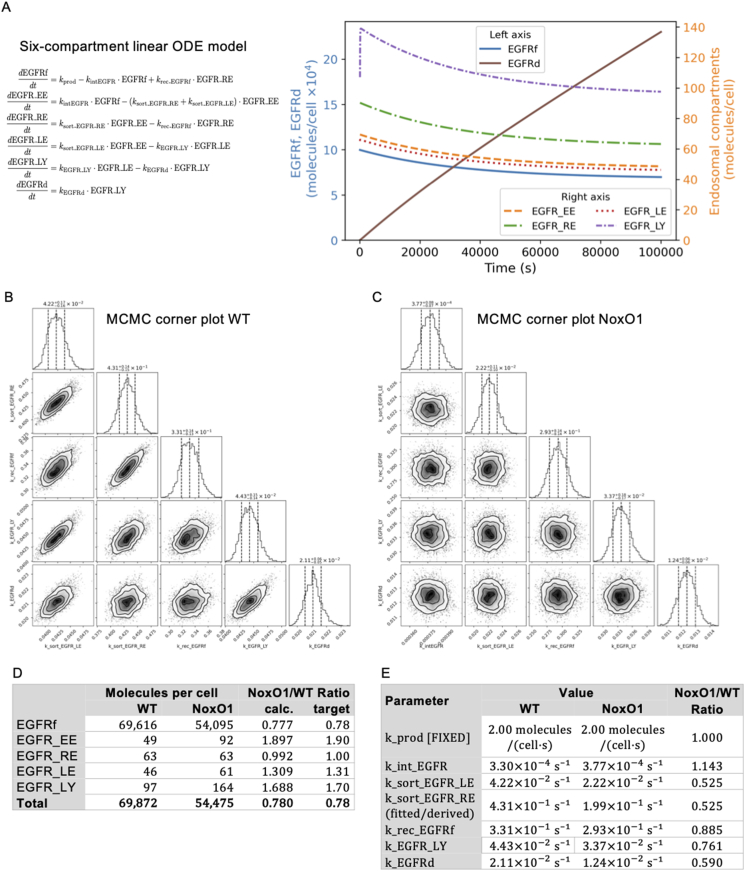
Simulation of EGFR degradation and Lysosome formation. (A) EGFR trafficking and degradation simulated using a six-compartment linear ODE model (EGFRf, EGFR_EE, EGFR_RE, EGFR_LE, EGFR_LY, EGFRd) solved numerically. Time evolution of EGFR compartment populations for the calibrated wild-type (WT) model, approaching steady state, using the fitted trafficking rates. (B&C) MCMC (emcee EnsembleSampler) fitting simulated to observed ratios; lysosomal volume/number scaling was included as an inferred parameter to predict the lysosome formation/volume change required to reproduce observed EGFR degradation. Corner plot of MCMC posterior distributions for wild-type (WT in (B)) and NoxO1 overexpression (NoxO1 in (C)) EGFR trafficking parameters. Diagonal panels show marginal distributions for each parameter; off-diagonal panels show pairwise joint distributions, revealing correlations. The parameters given at the top of each distribution are the median value and the 1σ credible interval. (D) Comparison of steady-state compartment levels and (E) comparison of transfer parameter constants between WT and NoxO1 overexpressing models.

As EGFR and EGF represent a well-studied signaling pathway involving endosomal dependent receptor turn over and cytokine signaling, we utilized this pathway as endogenous cellular model for intracellular trafficking. We created a model of EGFR abundance in each state on its intracellular journey based on the available literature (Fig. 2A) [11,23,24].

We calibrated a six-compartment ordinary differential equation model of EGFR trafficking using the Markov Chain Monte Carlo (MCMC) method. For the basal condition, we fitted production rate and five transfer rate constants to six experimental constraints derived from Fig. 1. MCMC proved essential for finding an optimal compromise that best satisfies all constraints simultaneously, yielding a steady-state distribution where 99.6 % of receptors reside on the cell surface and the remaining 0.4 % are distributed across endosomal compartments. The calibration for the effect of NoxO1 overexpression, constrained by experimental NoxO1/WT ratios for compartment levels, revealed that cells adapt to NoxO1 overexpression through a coordinated set of trafficking changes: internalization rates increase by 14 % while degradative sorting decreases by 48 %, lysosomal transfer slows by 24 %, and final degradation rates decline by 41 % (Fig. 2B–E). These compensatory adjustments result in receptor accumulation within endosomal compartments (early endosomes increase 1.9-fold, late endosomes 1.3-fold, and lysosomes 1.7-fold), suggesting that cells actively modulate trafficking dynamics in response to NoxO1 overexpression. As previously mentioned, the decrease in total cellular receptor abundance is at the detection limit. In order to maintain the observed equilibrium of EGFR abundance, the intracellular trafficking of endocytosed cargo must be slowed down, resulting in stalling of the cargo in lysosomes. Accordingly, the results suggest that lysosomes are overfilled with membrane-born cargo.

### 2.3 Overexpression of NoxO1 results in overload of lysosomes with cargos

**Fig. 3:**
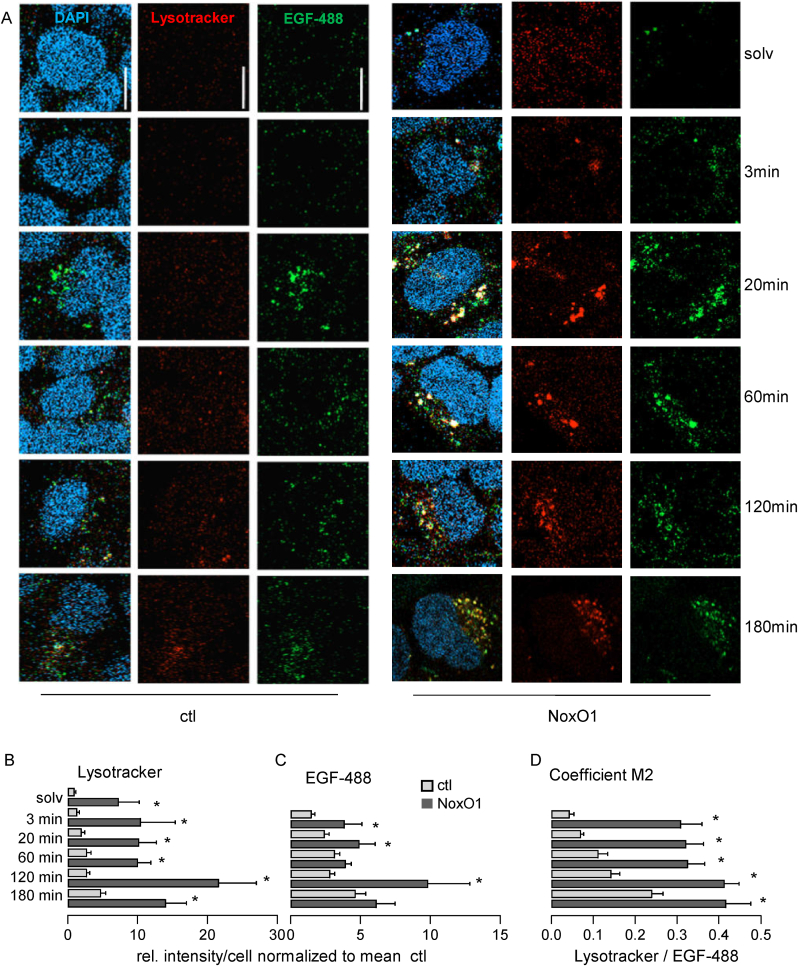
NoxO1 promotes EGF internalization and translocation into lysosomes in Hek293. Cells overexpressing empty vector (ctl) or NoxO1 (NoxO1) were treated with EGF-488 [50 ng/mL] or solvent (solv) for the indicated times. All samples were co-incubated with Lysotracker Red [50 nM]; ∗p < 0.05 in two-sided student's *t*-test for each condition ctl vs. OE. Mean ± SEM, n = 3–6; (A) Representative images, scale bar = 10 μm; (B) Lysosome formation was quantified by Lysotracker Red; (C) Intracellular EGF-488; (D) Lysotracker Red-EGF-488 colocalization analyzed by Manders Coeffcient M2, which determines the fraction of EGF-488 overlapping with Lysotracker Red.

For validation of this prediction, cells were treated with fluorescently labeled EGF and uptake of the growth factor was followed along with observation of lysosome architecture. Besides more lysosomes in NoxO1 overexpressing cells, more EGF was found to end up in the lysosomes (Fig. 3). Actually, the number of EGF located in lysosomes was increased, when compared to the control. Since NoxO1 increases lysosome formation, intracellular vesicle transport, and maturation, we thought this effect may not be EGF specific, but rather represent a general mechanism that should also be observable with other cargoes, such as BSA. We treated the cells with fluorescently labeled BSA as we did with EGF. Unlike EGF, BSA uptake was much slower and less effective. However, as seen with EGF NoxO1 overexpression, resulted in a higher proportion of BSA co-localized with lysosomes when compared to control cells (Supplemental Fig. S3). Since endosomal trafficking of BSA is presumably not linked to a specific receptor or endocytosis pathway [16], NoxO1 may engage with endosomal transport and fate of multiple cargoes. We conclude a larger pool of preformed lysosomes enhances endosomal maturation and cargo trafficking irrespective of the cargo's specific kinetics. Additionally, this finding also confirms that we are not observing an unfolded protein response (UPR) induced by overexpression, as lysosomes would primarily contain the overexpressed protein rather than serve as active components of the endosomal trafficking machinery [25].

### 2.4 NoxO1 activates TFEB via Erbin without contribution from Nox1-derived ROS

**Fig. 4:**
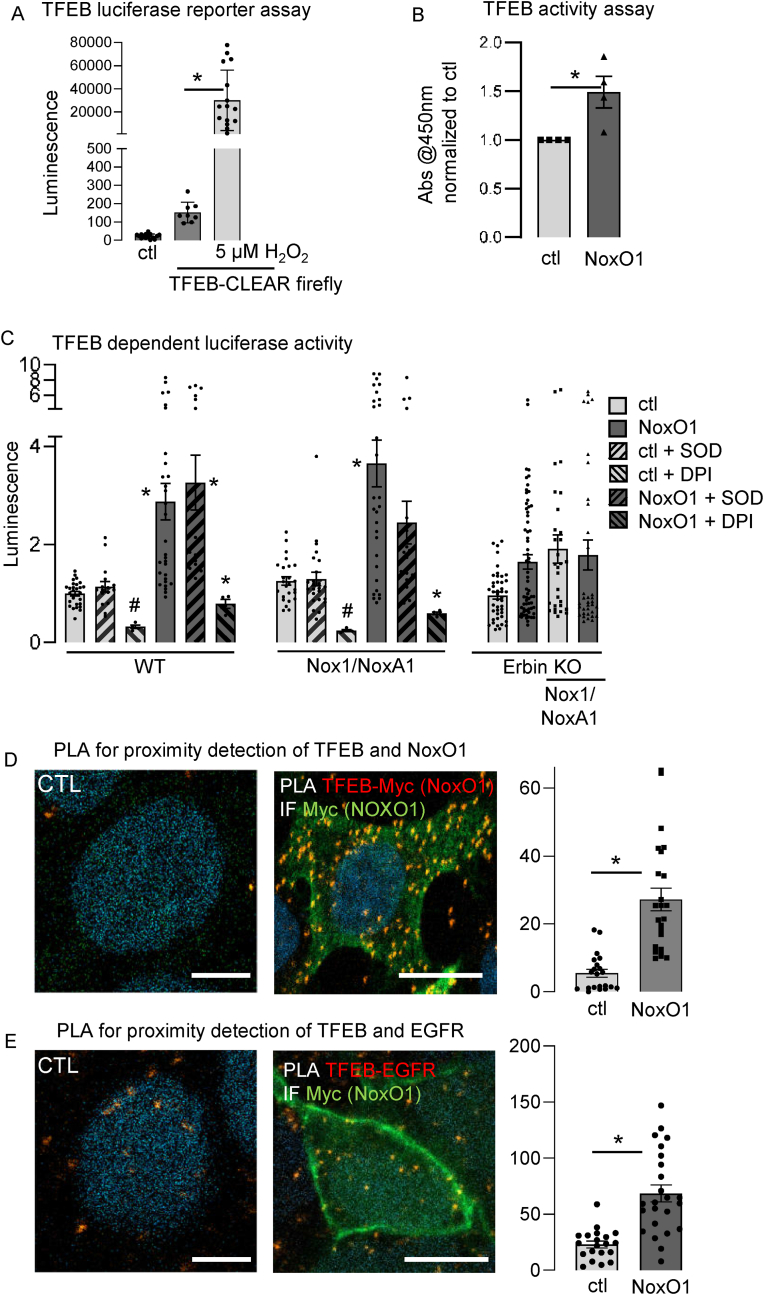
NoxO1 regulates nuclear TFEB activity. (A) Luciferase reporter gene assay in Hek293 cells without or with overexpression of TFEB-CLAER firefly ® treated without or with 5 μm H2O2. (B) TFEB activity assay kit® with 15 μg nuclear protein extracts. Cells overexpresssing either NoxO1 (NoxO1) or an empty vector (ctl). Readout at 450 nm absorbance. n = 3–4, ∗p < 0.05, One Way ANOVA + Tukey post hoc test. Mean ± SEM; (C) Luziferase reporter gene assay in wildtype Hek293 cells (WT), Hek293 stably overexpressing Nox1 and NoxA1 (Nox1/NoxA1) and Erbin knock out cells (Erbin KO) with overexpression of TFEB-CLAER firefly ® treated with or without SOD [50U/ml] or DPI [1 μM]. n = 8–12, ∗p < 0.05 ctl vs. NoxO1, #p < 0.05 untreated vs. treated, *t*-test. Mean ± SEM; (D&E) Proximity ligation assays for interaction of (C) TFEB with NoxO1 and (D) TFEB with EGFR without and with overexpression of NoxO1.

**Fig. 5:**
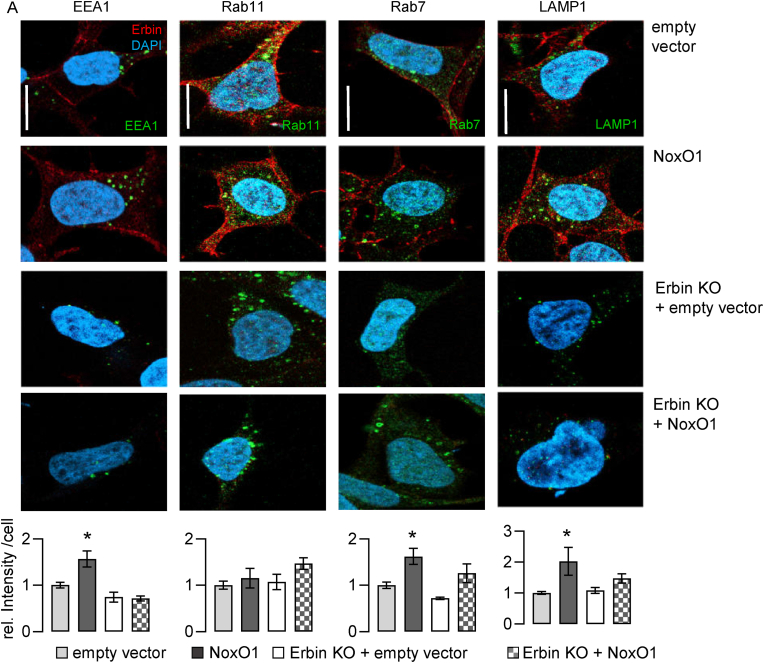
NoxO1 and Erbin cooperatively regulate endosomal pathways in Hek293 cells. Analyses of endosomal markers in resting cells with or without Erbin knock out (Erbin KO) overexpressing an empty vector or NoxO1 (OE): Early endosome antigen 1 (EEA1) for early endosomes, n = 6; Ras-related protein 11 (Rab11) for recycling endosomes, n = 6; Ras-related protein 7 (Rab7) for late endosomes, n = 3; Lysosome associated membrane protein 1 (LAMP1) for lysosomes, n = 4, ∗p < 0.05, ∗∗p < 0.01, ∗∗∗p < 0.001 in two-sided student's *t*-test for each condition or One Way ANOVA + Tukey post hoc test; mean ± SEM.

Instead, we found that transcription factor EB (TFEB), a master regulator of lysosome formation, was more active in cells overexpressing NoxO1. Martina et al. found TFEB to be activated by oxidation of cysteine 284 in *C. elegans* [26]. A luciferase assay confirmed ROS-dependent activation of TFEB in Hek293 cells (Fig. 4A). Surprisingly, TFEB nuclear activity was enhanced in cells solely overexpressing NoxO1 (Fig. 4B). In cells capable of forming a functional Nox1 complex (constitutive expression of Nox1/NoxA1 with NoxO1 overexpression), antioxidants reduced lysosome formation to the same level as seen with NoxO1 overexpression in cells not capable to form more Nox1 complex. Further, in the case of NoxO1 overexpression alone, neither antioxidants nor NADPH oxidase inhibitors were able to prevent an increase in lysosome formation (Supplemental Fig. S4). We conclude that ROS may induce TFEB activity, which subsequently elevates lysosome formation. Notably, NoxO1 can also induce this process independently of the other components of the Nox1 complex. Indeed, overexpressing NoxO1 in wild-type or Nox1/NoxA1 Hek293 cells increased TFEB activity to the same level. Interestingly, treating the cells with superoxide dismutase (SOD) to reduce Nox1-complex-derived superoxide, prevented a significant induction of TFEB activity. This suggests that in the presence of a functional Nox1 and NoxA1, NoxO1 prefers those as binding partners and contributes to ROS formation rather than interacting with TFEB. Diphenylene iodonium (DPI), which inhibits all flavoproteins and prevents nearly all cellular ROS formation, reduced the basal TFEB activity, but could not prevent the NoxO1 induced increase in TFEB activity (Fig. 4C). We previously identified Erbin as an interaction partner of NoxO1 [8]. Interestingly, knocking out Erbin prevented TFEB activity, regardless of the presence or absence of Nox1 and NoxA1. This indicates that Erbin is involved in NoxO1-induced elevation of TFEB activity (Fig. 4C). We conclude Erbin and NoxO1 act in concert to induce TFEB activity. Since NoxO1 can serve as a multimodal adapter protein we analyzed for its physical proximity with TFEB. Due to the lack of a reliable NoxO1 antibody, we overexpressed a NoxO1-myc fusion protein for further analysis and found an association of NoxO1-myc and TFEB (Fig. 4D). An earlier study has described a correlation between the expression of EGFR and TFEB [27]. As Erbin engages with EGFR, we hypothesized that EGFR may facilitate the proximity of Erbin to TFEB. This hypothesis was subsequently validated by the observation of EGFR in close proximity to TFEB (Fig. 4E). Analysis of markers indicative of organelles in the endosomal pathway revealed that NoxO1 and Erbin cooperate to regulate the expression of these markers. Knockout of Erbin prevented the NoxO1-induced elevation in EEA1 and LAMP1 expression (Fig. 5).

Together these data suggest the existence of a NoxO1/Erbin/EGFR/TFEB complex responsible for enhanced lysosome biogenesis.

## 3 Discussion

In this study, we identified a novel function for NoxO1 in controlling endosomal trafficking and lysosome biogenesis. NoxO1 overexpression increases early endosome and lysosome abundance while simultaneously slowing cargo transit through the endolysosomal system, resulting in prolonged retention of both membrane-bound and soluble macromolecules in lysosomes. Mechanistically, we demonstrate that NoxO1 activates the master regulator of lysosomal biogenesis, transcription factor EB (TFEB), via its interaction partner Erbin, and that this activation occurs independently of the canonical Nox1 complex and the ROS it produces. The precise mechanism through which NoxO1 modulates TFEB activity requires further elucidation in subsequent studies.

MAP kinases, including Erk and Akt, have been demonstrated to maintain TFEB in an inactive, phosphorylated state [28]. Shuttling between nucleus and cytosol controls TFEB activity [29] and Erbin was found to bind and control TFEB spatial distribution and activity [30]. Consequently, a model in which NoxO1 assists TFEB by maintaining Erbin in proximity to the transcription factor may provide a rational explanation for the observed effects. Prevention of negative aging related effects are facilitated by effective lysosome-mediated proteostasis [31], suggesting that a decrease in lysosome numbers, e.g. due to the loss of NoxO1, may accelerate the aging process. Notably, NoxO1 knockout mice have been observed to exhibit prolonged lifespans in comparison to their wild-type counterparts [32]. While the present study concentrated on the investigation of cellular lysosomes, the absence of NoxO1 has the potential to influence lysosome quantity and function at the organism level. Preliminary research has demonstrated that NoxO1 exerts sex-dependent effects. For instance, female NoxO1 knockout mice exhibit a protective response against atherosclerosis when fed a high-fat diet, while male counterparts do not demonstrate a similar response ([6] and unpublished observation). Lysosomes play a crucial role in regulating cholesterol homeostasis and dysfunctional lysosomes have been shown to impair cholesterol degradation [33]. Especially in the context of atherosclerosis, disparities in cholesterol metabolism may influence the capacity of macrophages to transform into foam cells [34]. In fact, lysosomes contribute to the clearance of lipids and cellular debris from macrophages, which is crucial in preventing the formation of foam cells during atherosclerosis [35]. The enhancement of lysosomal function in the absence of NoxO1 may facilitate the clearance of these materials, thereby reducing plaque formation and enhancing cardiovascular health. Consequently, it is imperative to deliberate the prospective repercussions of sex hormones in subsequent research endeavors concerning NoxO1 and its influence on lysosomes. The role of sex hormones in this context remains to be discovered.

A notable finding is that men exhibit a higher prevalence of certain cancers, such as colon cancer, compared to women [36]. Correct turnover of proteins and cytokines, along with proteostasis, may shield cells from becoming cancerous from malignant transformation [37]. The Kaplan-Meyer plot database (//kmplot.com/) provides publicly available data on gene expression in human breast and colon cancer, which reveals that patients with high NoxO1 expression in their tumors have improved overall and relapse-free survival (Supplemental Figure S5 A&B). While the investigation did not ascertain whether high NoxO1 expression in cancer increases lysosomal biogenesis as well, such an effect may help to sensitize cancer cells to chemotherapy [38,39]. Indeed, there are several potential medical applications for enhancing lysosome activity or increasing lysosome numbers in human patients. Lysosomes play a critical role in cellular housekeeping, including the degradation of misfolded proteins and damaged organelles [40]. Impaired lysosomal function is associated with the accumulation of toxic proteins, as seen in neurodegenerative diseases like Alzheimer's and Parkinson's [41,42]. Dysregulation of lysosomal function has been implicated in the development of metabolic disorders, including obesity and type 2 diabetes [43]. Eventually, lysosomal storage disorders, including Gaucher disease and Fabry disease, are eventually triggered by the absence of particular lysosomal enzymes, leading to the accumulation of undigested substrates [44].

The potential for leveraging lysosomal function to achieve therapeutic benefits in diverse medical contexts is a promising area of research. The augmentation of lysosome numbers through the activation of NoxO1 could offer a novel approach to enhance the clearance of debris and aggregates. Consequently, the identification of a small molecule inhibitor of NoxO1 may emerge as a promising therapeutic approach for a variety of diseases.

Limitation:

While our data clearly demonstrate that NoxO1 forms a functional scaffold with Erbin and TFEB, the precise stoichiometry and potential co-trafficking with EGFR/HER2 cannot be determined in the current study. Future work will address these important mechanistic questions.

## 4 Materials and methods

If not stated otherwise, human genes and proteins are addressed.

### 4.1 Cell lines and cell culture

Human cell lines (Hek293, CaCo2, and MCF-7) were purchased from ATTC (Manassas, USA) and cultivated in Minimal Essential Medium (MEM, #11095080 Gibco) with 1 mM Sodium pyruvate (#M7145 sigma), 0.1 mM Non-essential Amino acids (#S8636,sigma), 0.5 % Penicillin-Streptomycin (#15140-122 sigma) and 8–20 % fetal calve serum (FCS, #f7524 sigma). For MCF-7 cells, 0.01 mg/ml human insulin (#I9278, sigma) was supplemented. Cells were cultured under 5 % carbon dioxide atmosphere at 37 °C.

### 4.2 Overexpression systems

For transient overexpression, transfection was carried out with 1 μg/ml polyethyleneimine (PEI, #408727 sigma) or the Lipofectamine3000® Kit (#L3000001 Invitrogen) for 4–6 h at 37 °C in MEM without supplements. After exchange of MEM to growth media (see section cell lines), overexpression was allowed for 1 day before performing experiments.

Constitutive overexpression was generated by lentiviral transduction followed by selection with 400 μg/ml Hygromycin (#ALX-380-309-G001 Enzo) or 2 μg/ml Puromycin (#0240.4 Carl Roth). Lentiviral particles were produced in Lenti-X™ 293T cells (purchased from Takara) by transfection with 1 μg/ml PEI together with the packaging plasmids psPAX2/pmD2.G (#12260, #12259 Addgene) and the encoding plasmid. After 1–2 days, lentiviral particles were harvested from the supernatant and tested with Lenti-X™ GoStix™ Plus (#631280 Takara). Host cells were infected with 1 ml supernatant and 8 μg/mL Polybrene (#TR-1003-G Merck) for 1 day. Selection was started after 1–2 days. As control, cells were transduced with an empty vector construct (Table 1). All plasmids were verified by Sanger sequencing at Microsynth Seqlab GmbH (Göttingen, Germany).

**Table 1:** Overexpression systems.

protein expressed		backbone	tag for detection
Nox1	transient	pCMV.6-entry	c-myc, Flag-DDK
NoxA1			
NoxO1			
eGFP		pEGFP-C1	GFP
(empty vector)	constitutive	pLV-EF1a-IRES-Hygro	–
NoxO1			
Nox1+NoxA1		EF1aFull-hOct4-F2A-hKlf4-IRES-hSox2-P2A-hcMyc-W-loxP	
Renilla	transient	pGL.4	–
CLEAR firefly			
Firefly empty vector

**Table 2:** Primary antibodies.

Target	host	manufacturer	Product Reference
EEA1	mouse	BD	#610457
EGFR	rabbit	Invitrogen	#PA 1–1110, #PA5-85476
Erbin	rabbit	Thermo Fisher	#PA566288
LAMP1	mouse	Abcam	#ab25630
myc-tag	goat	Bethyl/Biomol	#A190-104A
NoxO1	rabbit	Eurogentec	#2110891 (customized)
Rab11A	mouse	Abcam	#ab78337
Rab7A	mouse	Abcam	#ab50533
LC3 A/B	rabbit	Cell Signaling	#4108
beta-Actin	mouse	Sigma	#A1978
P62	rabbit	Enzo	#BML-PW9860-0100

**Table 3:** Secondary antibodies.

Target	label	host	manufacturer	Product Reference
Anti-goat	AF488	donkey	Invitrogen	#A11055
Anti-mouse	AF488 AF546 AF647	donkey	Invitrogen	#A21202 #A10036 #A31571
Anti-mouse IRDye®	680RD 800CW	donkey	LI-COR	#926-68072 #926-32212
Anti-rabbit	AF488 AF546 AF647	donkey	Invitrogen	#A21206 #A10040 #A31573
Anti-rabbit IRDye®	680RD 800CW	donkey	LI-COR	#926-68073 #926-32213

### 4.3 Gene knockout by CRISPR/CAS system

NoxO1 and Erbin were knocked out by applying the lentiviral CRISPR/CAS technique in MCF7 or Hek293 cells, respectively. Guide RNAs (gRNAs) were designed at the crispor.tefor.net platform (Table 4) and cloned into lenti CRISPRv2 (#52961 addgene) backbone through Golden Gate Assembly [45]. Briefly, sense and antisense gRNAs were annealed at 98 °C for 5 min and subjected to restriction and ligation in a thermocycler. PCR product was transformed into the *E.coli* DH5a strain and positive clones selected by Ampicillin resistance. Plasmids were isolated using the GeneJET Plasmid Mini Kit and sequenced at MicroSynth Seqlab GmbH (Göttingen, Germany). Production of lentiviral vectors and transduction were conducted like for overexpression systems. Gene knockout was verified by PCR, Western blot and ROS formation.

**Table 4:** gRNAs for CRISPR/Cas9 gene knockout.

gene	sense (5′-3′)	antisense (5′-3′)
*Erbin*	CACCGTTACAGCAGTTGCCCCCAG	AAACCTGGGGGCAACTGCTGTAAC

### 4.4 Genomic DNA isolation and PCR

Confluent cells were mechanically detached from a 24-well dish and incubated for 30 min at 56 °C at 800 rpm in warm lysis buffer (5 mM Tris-HCl pH 8.5 #AE15.3 Carl Roth, 10 mM NaCl #31434-5 KG-R sigma 0.2 % SDS #CN30.3 Carl Roth, 5 mM EDTA #ED-1KG sigma, 3 μg Proteinase K #P2305-25 MG sigma). Lysates were spin down and the supernatant including genomic DNA (gDNA) precipitated with isopropanol. After pelletizing and several washings with 70 % ethanol, gDNA was dried and resuspended in water. 100–300 ng gDNA were used for PCR with primers flanking the CRISPR target side (Table 5). PCR product was separated by electrophoresis in 1.5 % universal agarose (#BS20.46.1009 VWR) gel in Mini Plus Horizontal chambers (Carl Roth). Gels were stained with Roti-Stain® and visualized at the Gel Stick (Intas).

**Table 5:** Primers for CRISPR validation through PCR or sequencing.

gene	forward (5′-3′)	reverse (5′-3′)
*GAPDH*	TGGTGTCAGGTTATG CTGGGCCAG	GTGGGATGGGAGGGTGCTGAACAC
*Erbin*	TGCAGTCAAAGACACTTTGTGG	–

### 4.5 ROS measurement with chemiluminescence

Reactive oxygen species (ROS) measurements assessing superoxide were carried out with L-012 (8-Amino-5-chloro-2,3-dihydro-7-phenyl-Pyrido[3,4-*d*]pyridazine-1,4-dione).

Living cells were resuspended in HEPES-Tyrode buffer (137 mM NaCl #31434-5 KG-R, 2.7 mM KCl #P9333, 0.5 mM MgCl #M8266, 1.8 mM CaCl2 #C7902, 5 mM d-Glucose #16301, 0.36 mM NaH2PO4∗H2O # 106346, 10 mM HEPES # H-3375, all from sigma) containing 200 μM L-012 (#120–04891, WAKO Chemicals). ROS production of 100 000 cells was assessed by chemiluminescence at 37 °C in a 6-channel luminometer. For quenching of superoxide, 20U superoxide dismutase (#S7571, sigma) was added.

### 4.6 Immunofluorescence and confocal microscopy

Immunofluorescence (IF) was performed on 8-well μ-slides (ibidi). After treatment, cells were fixed with Roti® Histofix (Carl Roth), washed with Dubecco's Phosphate-buffered saline (DBPS, #14040133 Gibco) and 2 % l-glycine (#A1377,5000 AppliChem). Cells were permeabilized with 0.05 % Triton-X 100 (Carl Roth). Unspecific binding sites were blocked with 3 % bovine serum albumin (BSA, #A8412, sigma). Primary antibodies (Table 2) were incubated (1:200) overnight and stained with AlexaFluor-conjugated secondary antibodies (1:500) (Table 3). Nuclei were stained with 0.1 μg/ml DAPI (4′,6-diamidino-2-phenylindole, #D9542 sigma). Slides were stored in the dark until detection with a confocal laser scanning microscope (LSM800, Zeiss). Other fluorescent probes comprised EGF-AF488™ (#E13345 Invitrogen), Lysotracker™Red DND-99(#L7528 Thermo Fisher) and BSA-AF488 (#13100, Invitrogen). Cells were stimulated with the probe at 37 °C as indicated in the result section and sample preparation was performed like an IF.

### 4.7 Proximity Ligation Assay

Proximity Ligation Assay (PLA) to visualize protein-protein interactions was performed using the Duolink® In situ Orange Kit (#DUO92007 sigma) according to the manufacturer's protocol.

Briefly, samples were prepared like for IF with the 2 primary antibodies against the interacting proteins (1:500 each). Secondary antibodies matching the antibody species and carrying oligonucleotides were ligated and amplified in a rolling circle reaction. Nuclei were stained with 0.1 μg/ml DAPI (4′,6-diamidino-2-phenylindole, #D9542 sigma). PLA signals were imaged with a laser scanning microscope (LSM800, Zeiss). Fluorescence was excited at 554 nm. PLA was combined with IF by co-incubation of primary antibodies (1:300) with the IF antibody for a third target. Secondary antibody for IF (1:500) was added after the last PLA polymerization step.

### 4.8 SDS-PAGE and western blot

Cells were lysed in TritonX-100 lysis buffer (250 mM Tris∗HCl pH7.4 #AE15.3 Carl Roth, 750 mM NaCl #S/3160/65 fisher, 50 mM NaPPi #106391 Merck, 100 mM NaF #201154 sigma, 10 % Triton-X #3051.3 Carl Roth, 2 mM Orthovanadate #A2196 AppliChem, 10 mM Okadaic Acid #ALX-350-011 Enzo, 200 μM PMSF #6367.1 Carl Roth, 20 μM cOmplete #4693116000 Merck) on ice. Samples were centrifuged (13000 rpm, 4 °C) and pellets were discarded. Total protein amount in the supernatant was quantified by spectrophotometric Bradford Assay with Roti-Quant®. Samples were boiled in *Laemmli* buffer at 95 °C. Sodium-Dodecylsulfate-Polyacrylamide-Gel-Electrophoresis (SDS-PAGE) was used to separate proteins on 10 % acrylamide gels followed by Western Blot using the MiniProtean system (BioRad). Unspecific binding sites were blocked with Roti-Block ®and primary antibodies (Table 2) incubated overnight (1:1000) at 4 °C. Membranes were incubated with secondary antibodies (1.15 000) labeled with IRDye® (Table 3) and scanned at an Odyssey (LI-COR).

### 4.9 TFEB activity (ELISA)

Transcription Factor EB (TFEB) activity was analyzed by using the respective Activity Assay Kit (#TFEH-TFEB-1, RayBiotech) according to the manufacturer's manual. In short, nuclear extracts of confluent cells were prepared (see section nuclear extraction) and 15 μg of nuclear proteins transferred to the ELISA microplate coated with TFEB-binding DNA sequences. After overnight incubation at 4 °C, primary antibody against TFEB was added for 1h followed by incubation with HRP-conjugated secondary antibody for another 1 h. TMB One-Step substrate was applied for 30 min and the enzymatic reaction was stopped with Stop solution. Absorbance was immediately measured at 450 nm. Controls were provided by the Kit (positive, non-specific and specific competitor).

### 4.10 Reporter gene assay (luciferase)

Binding of TFEB to its target genes was assessed by applying a Luciferase-based reporter gene assay. The Bright Glo™ Luciferase Assay System (#E2610, Promega) was performed according to the manufacturer's protocol.

Cells were cultured on white 96-well plates with flat transparent bottom and transfected with reporter plasmids either coding for the CLEAR promoter sequence coupled to firefly luciferase (#66800, addgene) or constitutively active renilla luciferase or empty PGL4-vector backbone.

Cells were allowed to overexpress these constructs for 24h and co-treated with DPI (#43088, Sigma) or SOD (#S7571, Sigma) to inhibit ROS production. Bright-Glo™ Luciferase Assay Substrate was reconstituted in Bright-Glo™ Luciferase Assay Buffer in the dark. The mixture was directly added to culture medium at room temperature and incubated for 5 min without prior cell lysis. Luminescence was measured immediately at a plate reader (Tecan).

### 4.11 Autophagy markers

P62 and LC3 served as autophagy markers in Hek293. Cells overexpressed ascending amounts of GFP or NoxO1. Expression of p62 and LC3 in unstimulated cells was determined by Western blot (see section Western blot). Treatment with 100 nM Bafilomycin A1 for 4h in full medium served as inhibitor of autophagy.

### 4.12 Lysosome formation and colocalization studies

Fluorescent probes comprised 50 ng/mL EGF-AF488™ (#E13345 Invitrogen), Lysotracker™Red DND-99(#L7528 Thermo Fisher) and 50 μg/mL BSA-AF488 (#13100, Invitrogen). Cells were stimulated with the respective probe at 37 °C and sample preparation was performed like an IF (see section Immunofluorescence). In order to quench ROS formation, cells were co-incubated with the following antioxidants for 4h:

10 μM DPI (#43088, Sigma), 10 U SOD (#S7571, Sigma); 10 U Catalase (#), 500 μM Vitamin C (# A4544, Sigma), 50 μM Vitamin E (#T3126, Sigma), 75 μM β-Mercaptoethanol (#M3148, Sigma), 2 mM Glutathione (#GU626, Sigma), 100 μM Quercetin (#PHR1488, Sigma). Colocalization was analyzed by the automated Coloc2 Fiji plugin thereby providing Manders Coefficients M1 and M2.

### 4.13 Simulation of EGFR internalization, intracellular trafficking and degradation

We developed a six-compartment ordinary differential equation (ODE) model to describe EGFR trafficking dynamics under basal (unstimulated) conditions in Hek293 cells. The model is implemented in Python and uses standard libraries (SciPy) to solve the system of ODEs. It tracks receptor molecules through distinct cellular compartments: free surface receptors (EGFRf), early endosomes (EGFR_EE), recycling endosomes (EGFR_RE), late endosomes (EGFR_LE), lysosomes (EGFR_LY), and a cumulative pool of degraded receptors (EGFRd). Trafficking between compartments is governed by one production flux constant k_prod and six transfer rate parameter constants representing key cellular processes: constitutive production (k_prod [molecules/(cell·s)]), internalization (k_int_EGFR [s^−1^]), sorting from early endosomes to either recycling (k_sort_EGFR_RE [s^−1^]) or degradative (k_sort_EGFR_LE [s^−1^]) pathways, recycling to the surface (k_rec_EGFRf) [s^−1^], transfer from late endosomes to lysosomes (k_EGFR_LY [s^−1^]), and proteolytic degradation (k_EGFRd [s^−1^]). Initial values were extracted from the literature [11,23,24,45]. The system of ordinary differential equations was integrated numerically using the BDF (Backward Differentiation Formula) method implemented in SciPy's solve_ivp function with relative and absolute tolerances of 10^−6^ and 10^−8^ respectively, ensuring accurate solutions for this stiff system. We employed Markov Chain Monte Carlo methods within a Bayesian inference framework to estimate model parameters from experimental constraints. For the wild-type calibration, we fixed two parameters based on independent measurements (k_int_EGFR = 3.3 × 10^−4^ s^−1^ and k_prod = 2.0 molecules/(cell·s) from steady-state mass balance) and fitted the remaining five rate constants to six experimental constraints representing steady-state compartment ratios and absolute receptor counts. MCMC was essential for the wild-type case because the experimental constraints proved mutually inconsistent: measurements of surface fraction and total receptor number imply endosomal compartments should contain approximately 10 500 molecules, while measurements of inter-compartmental ratios predict only 219 molecules, a 48-fold discrepancy that precludes algebraic solution. The MCMC sampler (emcee ensemble sampler with 16 walkers run for 1000–2000 steps with 250–750 step burn-in) finds the globally optimal parameter values that best satisfy all constraints simultaneously, with each constraint weighted by its experimental uncertainty. Likelihoods were computed in log-space to appropriately handle relative errors in ratio measurements, and uniform priors spanning 2-4 orders of magnitude were imposed to ensure physically plausible parameter values. For the overexpression calibration, we fixed k_prod at its wild-type value (reflecting the biological interpretation that overexpression alters receptor stability rather than biosynthesis rates). The sorting ratio was assumed as k_sort_EGFR_RE = 9 × k_sort_EGFR_LE. The remaining five rate constants (k_intEGFR, k_sort_EGFR_LE, k_rec_EGFRf, k_EGFR_LY, and k_EGFRd) were fitted to five experimental constraints expressed as NoxO1/WT ratios for compartment levels. Unlike the wild-type case, the overexpression system proved nearly exactly determined with minimal constraint inconsistency, such that an algebraic solution exists and matches numerical solutions to within 2 %. Nevertheless, MCMC provided valuable refinement reducing errors below 1 % and, critically, quantified parameter uncertainties through full posterior distributions, enabling rigorous propagation of uncertainty to model predictions. All parameter estimates are reported as median values with 68 % credible intervals defined by the 16th and 84th percentiles of the posterior distribution.

### 4.14 Publicly available data

Kaplan Meyer Plots (KMP) were created by the public available platform Kaplan-Meier plotter (kmplot.com) for NoxO1 expression (235329_at). Analysis was not restricted to staging (lymph node status, grade), tumor subtypes (St.Gallen, PAM50) or receptor status (ER, PR, Her2, p53) either. Patients were split by median and redundant samples removed (51).

### 4.15 Statistical analysis

Data are presented as mean and standard error of the mean (SEM). All experiments were at least conducted in three independent biological replicates, defined by “n”. Calculations and statistical analysis were performed with Prism 10 (Graph Pad). p-values smaller than 0.05 were accepted as statistically significant. ∗p < 0.05. In case of multiple statistical tests, Tukey *post hoc* correction was applied. Normalizations are indicated in the graphs.

### 4.16 Data and code availability

All data supporting the findings of this study are available within the article and its Supplementary Information files. The EGFR trafficking model code, parameter files, and scripts used for calibration and analysis will be deposited in a public repository (GitHub) and made available without restrictions. Additional data generated and analyzed for the current study are available from the corresponding author upon reasonable request.

## Significance statement

Here we provide evidence that NoxO1 enhances endosome and lysosome formation, while delaying inter-organelle trafficking and cargo degradation, in an Erbin dependent manner. This study supports the role of NoxO1 as an adaptor protein with functions beyond the well-established enabling of Nox1 mediated ROS formation.

## CRediT authorship contribution statement

**Maureen Hebchen:** Formal analysis, Methodology, Visualization, Writing – original draft. **Falk Herwig:** Conceptualization, Formal analysis, Investigation, Methodology, Software, Visualization. **Tim Schader:** Investigation, Methodology. **Manuela Spaeth:** Investigation, Methodology. **Niklas Müller:** Methodology. **Katrin Schröder:** Conceptualization, Formal analysis, Funding acquisition, Project administration, Supervision, Visualization, Writing – original draft, Writing – review & editing.

## Declaration of competing interest

The authors declare no conflict of interest.

We confirm that neither the manuscript nor any parts of its content are currently under consideration or published in another journal. All authors have approved the manuscript and agree with its submission. We confirm that there are no image duplication, image manipulation, or visual plagiarism.

## Figures and Tables

**Table:** 

BSA	Bovine Serum Albumin
CHX	Cycloheximide
CQ	Chloroquine
DPI	Dipehyleneidonium
EEA1	Early Endosomal Antigen 1
EGF(R)	Epidermal Growth Factor (Receptor)
Erbin	Erbb Interacting Protein
GSH	Glutathione
Hek293	Human Embryonic Kidney cells 293
IF	Immunofluorescence
KO	Knockout
LAMP1	Lysosomal-associated membrane protein 1
NoxO1	NADPH oxidase organizer 1
OS	Overall Survival
PIM	Protease Inhibitor Mix
PLA	Proximity Ligation Assay
Rab7 (or 11)	Ras-related protein Rab 7 or 11
RFS	Relapse Free Survival
ROS	Reactive Oxygen Species
SOD	Superoxide Dismutase
TFEB	Transcription Factor EB
WB	Western Blot

## Data Availability

Data will be made available on request.
