# Supplementary material for: NoxO1 promotes endosome formation and reduces intracellular vesicle processing
Source: Redox Biol. 2025 Dec 12;89:103973. doi: 10.1016/j.redox.2025.103973 (PMC12808832; doi:10.1016/j.redox.2025.103973)
Supplement: Multimedia component 1 [file mmc1.pdf]

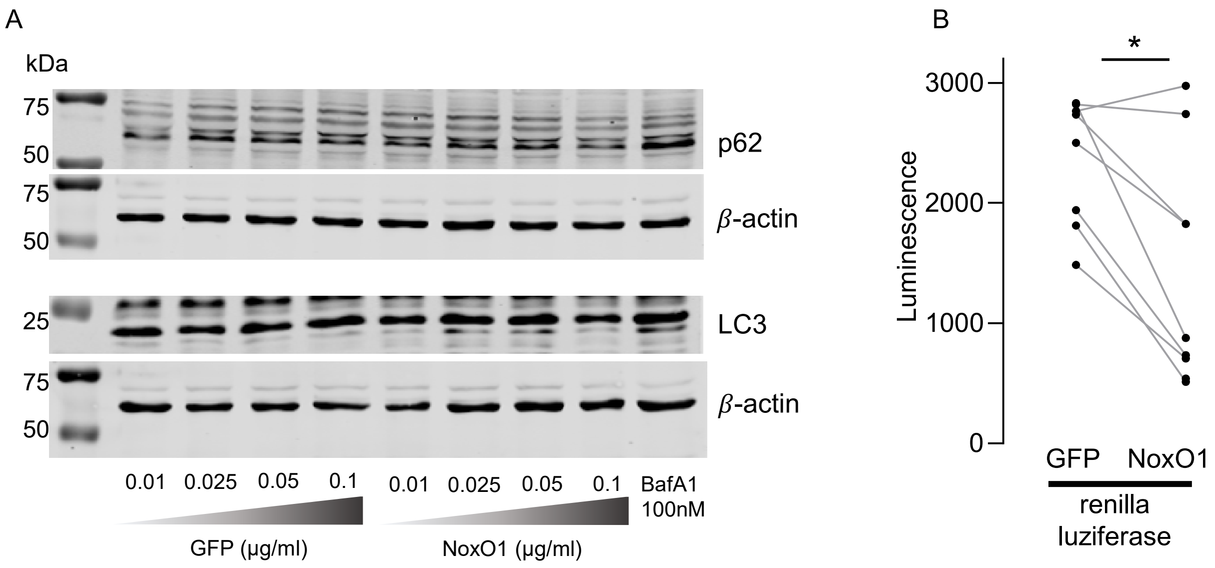

Supplemental Figure S1: NoxO1 overexpression in Hek293 cells forces protein degradation but not autophagy. (A) Western blot for autophagy markers (p62 and LC3) in Hek293 with overexpression of GFP or NoxO1 in ascending concentration; (B) Luziferase reporter gene assay in Hek293 overexpressing Renilla luciferase® and GFP or NoxO1. n=7, *p<0.05, paired t-test.

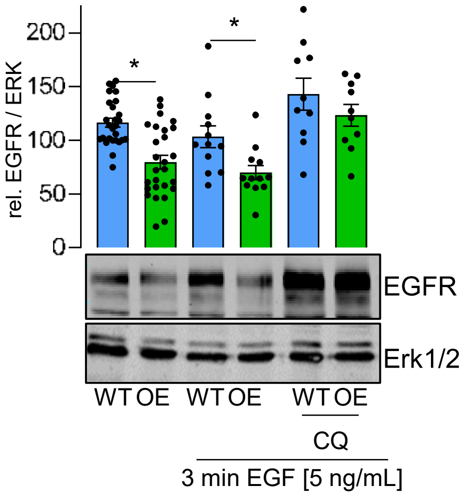

Supplemental Figure S2: NoxO1 overexpression in Hek293 promotes EGFR degradation. Wildtype (WT) cells and cells constitutively overexpressing NoxO1 (OE) were treated with EGF or solvent (solv). Stability assessment of EGFR protein level in presence of the translational inhibitor cycloheximide (CHX). Cells were pre-treated with or without the translational inhibitor cycloheximide (CHX) [10 µg/mL] for 1h and co-treated with EGF [5 ng/mL] w/o chloroquine (CQ) [200 µM]. Representative WB and Quantification of EGFR protein level, n = 10-27; *p<0.05 in two-sided student's t-test for each condition. Mean ± SEM

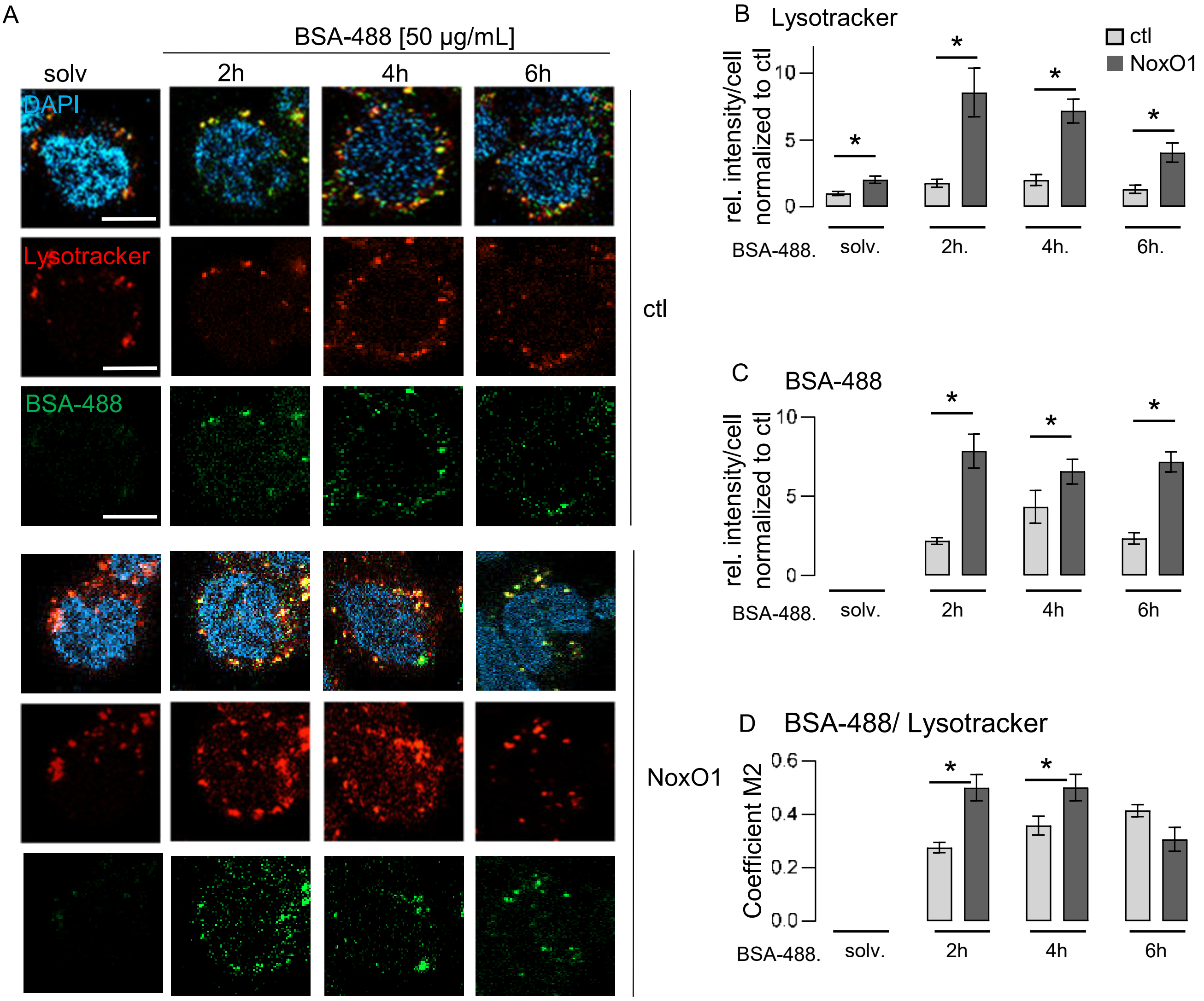

Supplemental Figure S3: NoxO1 promotes BSA internalization and translocation into lysosomes in Hek293. Cells overexpressing NoxO1 (OE) or empty vector (ctl) were treated with BSA-488 [50 µg/mL] or solvent (solv) for the indicated times. All samples were co-incubated with Lysotracker Red [50 nM]. *$p<0.05$, in two-sided student's t-test for each condition. Mean ± SEM, n=4

(A) Representative images, scale bar = 10 µm.

(B) Lysosomes identified by Lysotracker Red

(C) Intracellular BSA-488

(D) Lysotracker Red-BSA-488 Colocalization analyzed by Manders Coeffcient M2, which determines the fraction of BSA-488 overlapping with Lysotracker Red..

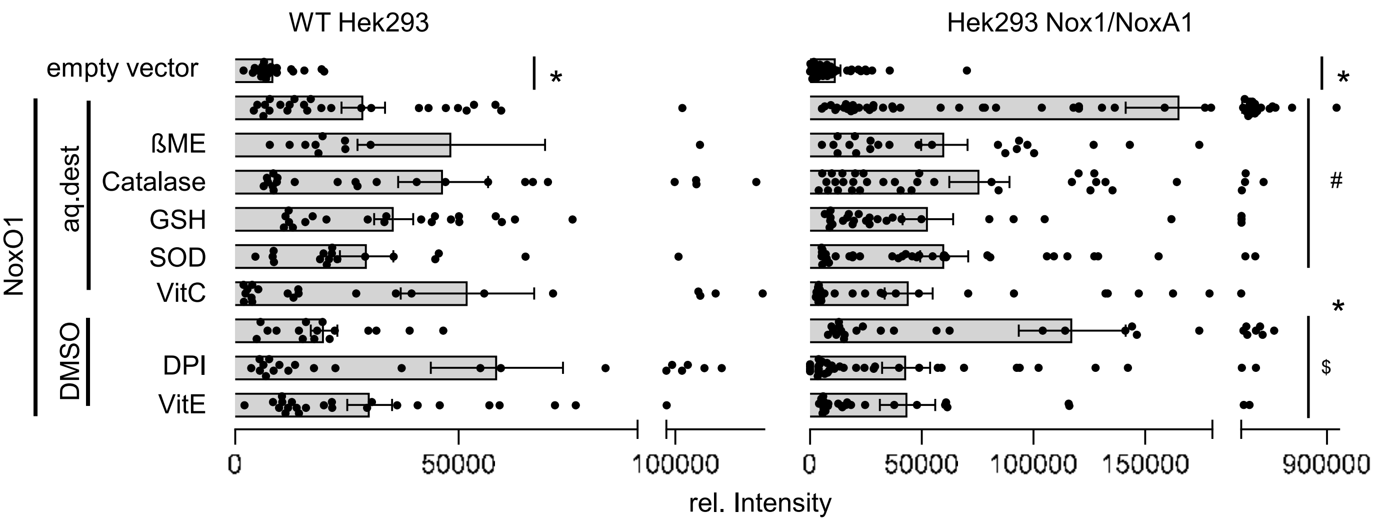

Supplemental Figure S4: NoxO1 overexpression in Hek293 cells forces lysosome formation in a ROS independent manner. (A) rel. intensity of lysotracker red as marker for active lysosomes in Hek293 (without (WT Hek293) or with stable overexpression of Nox1 and NoxA1(Hek293 Nox1/NoxA1)) with overexpression of NoxO1. Cells were treated with plain water (Aq. dest), ß-mercaptoethanol (ßME), Catalase, gluthathion (GSH), superoxide dismutase (SOD), vitamin C (VitC), DMSO, diphenyleneiodonium chloride (DPI), quercinin or vitamin E (VitE) n=5, *p<0.05 empty vector vs. NoxO1 overexpression, $/#p<0.05 NoxO1 overexpression/solvent vs. antioxidant as indicated , unpaired t-test.

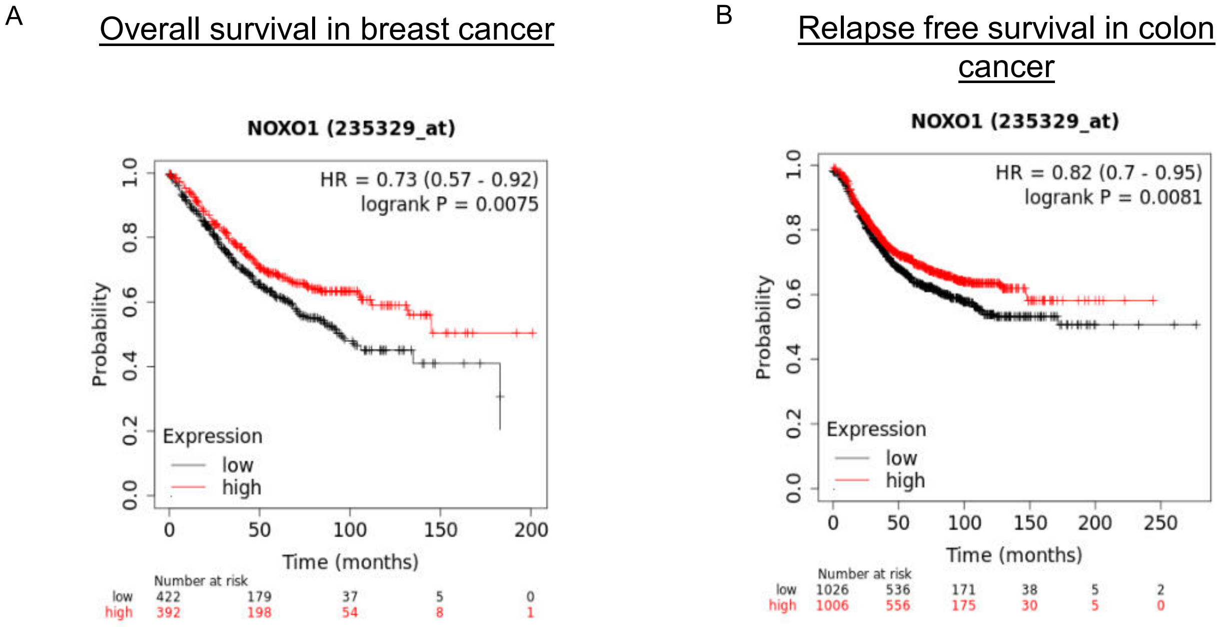

Supplemental Figure S5: Potential impact of NoxO1 on patients‘ outcome. Kaplan Meyer plots in breast cancer (A) and colon cancer (B) from publicly available data (//kmplot.com/), Györffy *et al*., 2021) without any restrictions of cohorts.

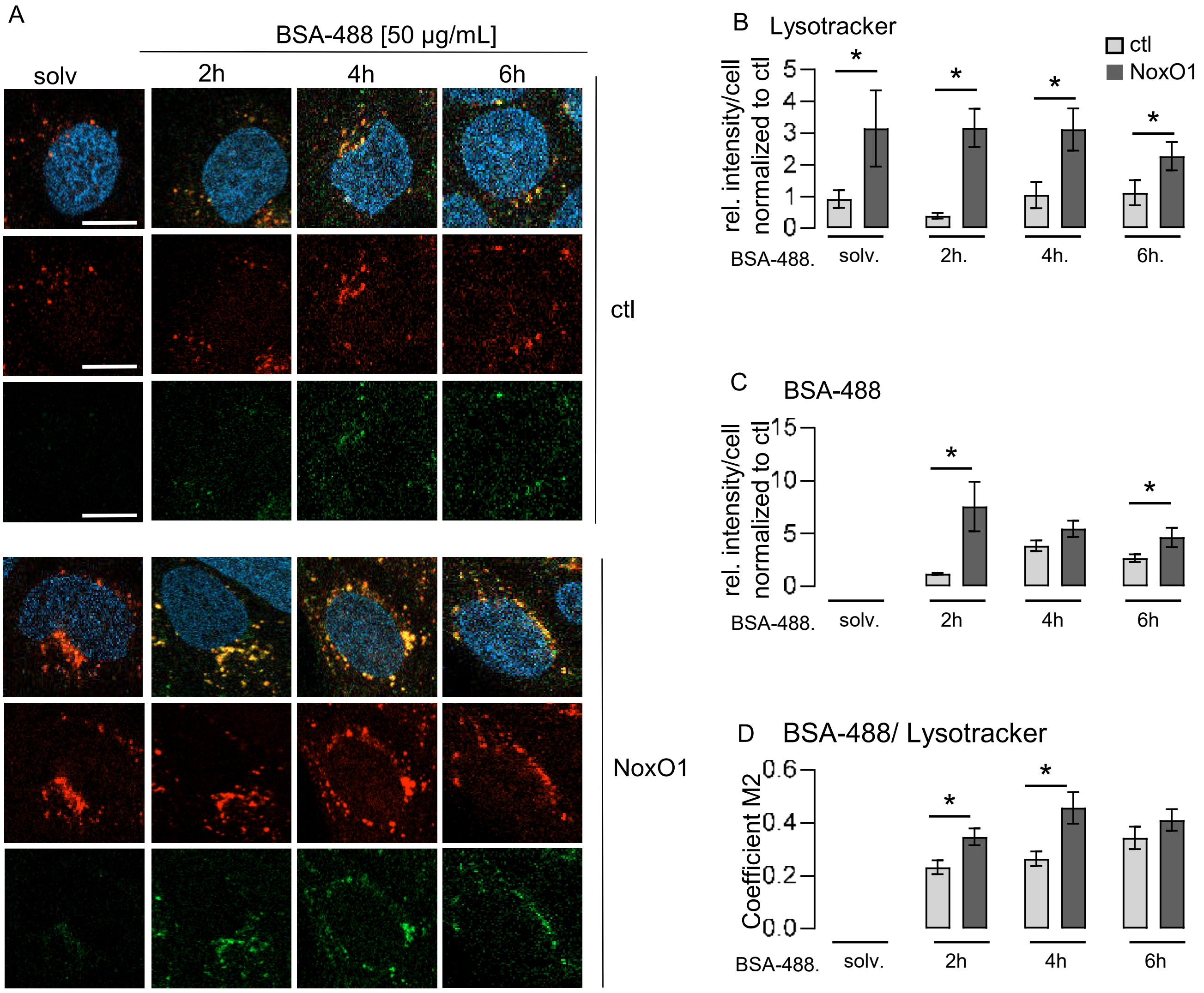

Supplemental Figure S6: NoxO1 promotes BSA internalization and translocation into lysosomes in CaCo2. Cells overexpressing NoxO1 or empty vector (ctl) were treated with BSA-488 [50 µg/mL] or solvent (solv) for the indicated times. All samples were co-incubated with Lysotracker Red [50 nM].]. *$p<0.05$, in two-sided student's t-test for each condition. Mean ± SEM, n=4

(A) Representative images, scale bar = 10 µm.
(B) Lysosome formation was quantified by Lysotracker Red.
(C) Intracellular BSA-488
(D) Lysotracker Red-BSA-488
(E) Colocalization analyzed by Manders Coeffcient M2, which determines the fraction of BSA-488 overlapping with Lysotracker Red.

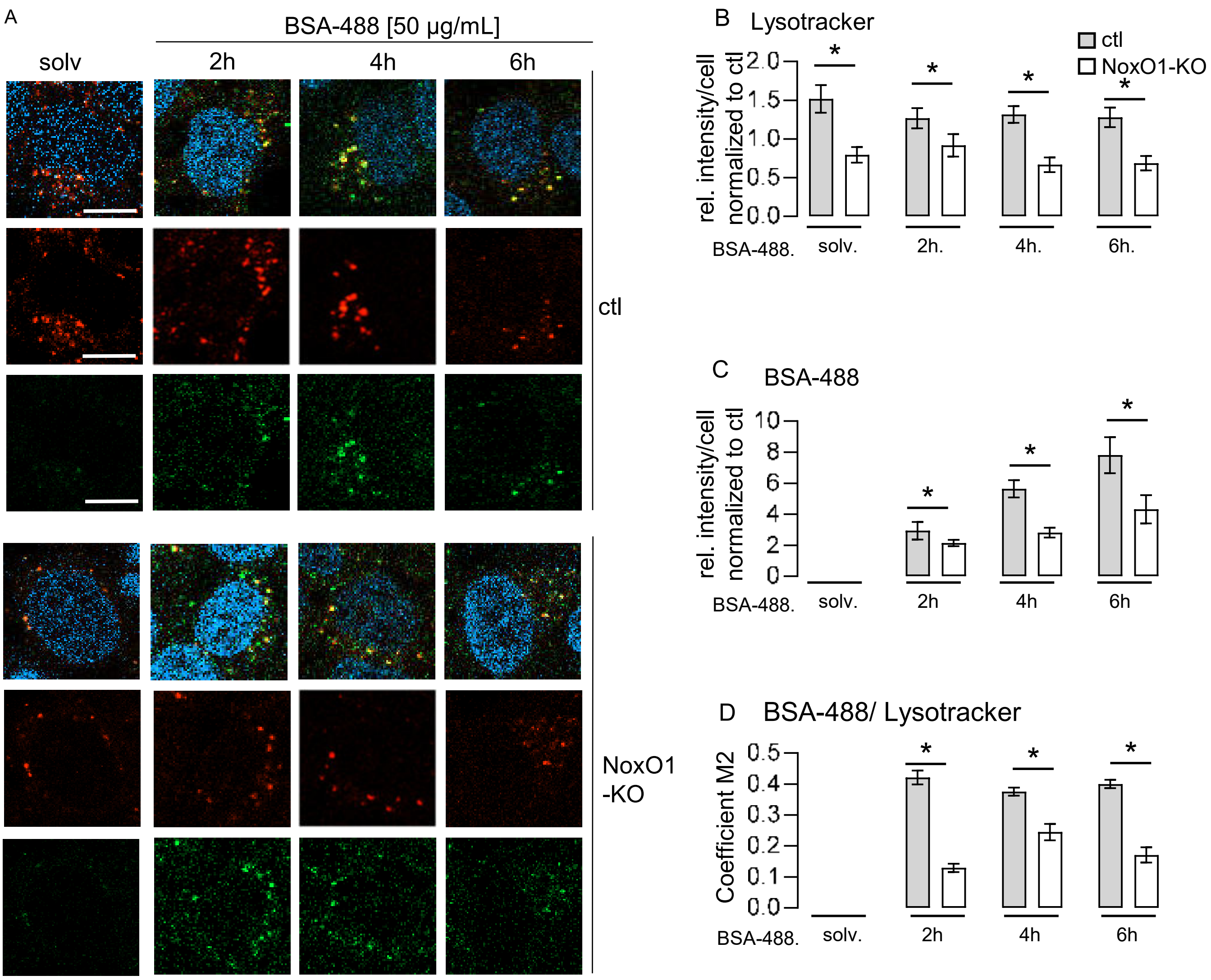

Supplemental Figure S7: NoxO1 promotes BSA internalization and translocation into lysosomes in MCF7. Cells with NoxO1 knock out (NoxO1-KO) or empty vector (ctl) were treated with BSA-488 [50 µg/mL] or solvent (solv) for the indicated times. All samples were co-incubated with Lysotracker Red [50 nM].*$p<0.05$, **$p<0.01$, ***$p<0.001$ in two-sided student's t-test for each condition. Mean ± SEM, n=4

(A) Representative images, scale bar = 10 µm.
(B) Lysosome formation was quantified by Lysotracker Red.
(C) Intracellular BSA-488 Lysotracker Red-BSA-488
(D) Colocalization analyzed by Manders Coeffcient M2, which determines the fraction of BSA-488 overlapping with Lysotracker Red.
